# ChIP-on-chip analysis identifies IL-22 as direct target gene of ectopically expressed FOXP3 transcription factor in human T cells

**DOI:** 10.1186/1471-2164-13-705

**Published:** 2012-12-17

**Authors:** Andreas Jeron, Wiebke Hansen, Franziska Ewert, Jan Buer, Robert Geffers, Dunja Bruder

**Affiliations:** 1 Immune Regulation group, Helmholtz Centre for Infection Research, Inhoffenstraße 7, Braunschweig, 38124, Germany; 2 Infection Immunology group, Institute of Medical Microbiology, Faculty of Medicine, Otto-von-Guericke University Magdeburg, Leipziger Straße 44, Magdeburg, 39120, Germany; 3 Institute of Medical Microbiology, University Hospital Essen, Hufelandstraße 55, Essen, 45122, Germany; 4 Genome Analytics group, Helmholtz Centre for Infection Research, Inhoffenstraße 7, Braunschweig, 38124, Germany

**Keywords:** FOXP3, ChIP-on-chip, Jurkat T cells, Transcription factor binding sites, IL-22

## Abstract

**Background:**

The transcription factor (TF) forkhead box P3 (FOXP3) is constitutively expressed at high levels in naturally occurring CD4^+^CD25^+^ regulatory T cells (nTregs). It is not only the most accepted marker for that cell population but is also considered lineage determinative. Chromatin immunoprecipitation (ChIP) of TFs in combination with genomic tiling microarray analysis (ChIP-on-chip) has been shown to be an appropriate tool for identifying FOXP3 transcription factor binding sites (TFBSs) on a genome-wide scale. In combination with microarray expression analysis, the ChIP-on-chip technique allows identification of direct FOXP3 target genes.

**Results:**

ChIP-on-chip analysis of the human FOXP3 expressed in resting and PMA/ionomycin–stimulated Jurkat T cells revealed several thousand putative FOXP3 binding sites and demonstrated the importance of intronic regions for FOXP3 binding. The analysis of expression data showed that the stimulation-dependent down-regulation of IL-22 was correlated with direct FOXP3 binding in the IL-22 promoter region. This association was confirmed by real-time PCR analysis of ChIP-DNA. The corresponding ChIP-region also contained a matching FOXP3 consensus sequence.

**Conclusions:**

Knowledge of the general distribution patterns of FOXP3 TFBSs in the human genome under resting and activated conditions will contribute to a better understanding of this TF and its influence on direct target genes, as well as its importance for the phenotype and function of Tregs. Moreover, FOXP3-dependent repression of Th17-related IL-22 may be relevant to an understanding of the phenomenon of Treg/Th17 cell plasticity.

## Background

To prevent inflammation-related collateral tissue damage and immune pathology caused by an excessive immune response to autoimmune-related self-antigens or pathogen-associated antigens, the immune system has developed a diverse spectrum of mechanisms allowing efficient regulation and suppression of innate and adaptive immune responses that maintain a homeostatic environment. One important cellular component mediating the control of immune responses is regulatory T cells (Tregs), which comprise various subpopulations. The so-called naturally occurring Treg cells (nTregs) have been described as being CD4^+^ T cells expressing high levels of the interleukin-2 receptor alpha-chain (known as CD25) and exhibiting immune-suppressive capability to self- and non-self-antigens [1]. The nTregs develop in the thymus along with conventional T cells and are considered to be the primary component for the establishment and maintenance of peripheral immunological self-tolerance [2]. Later it has been found that nTregs in mice can be identified by the expression of the transcription factor (TF) forkhead box P3 (Foxp3) [3].

Mutations in the human FOXP3 gene are linked to the Immunodysregulation polyendocrinopathy enteropathy X-linked syndrome (IPEX), which is accompanied by a defect in the development of nTregs. This defect affects the peripheral tolerance and surveillance of auto-reactive T cells, thereby promoting systemic autoimmunity [4].

Interestingly, most mutations can be mapped to the forkhead-binding protein domain (FKH) [5], which mediates the general interaction of FOXP3 with genomic DNA [6] and with FOXP3 transcription factor binding sites (TFBSs) in particular. TFBSs can be defined by consensus sequences that have been determined for FOXP3 in several studies. *De novo* identification of human FOXP3 consensus sequences revealed the sequence *5*^′^*-GTAAACAA-3*^′^[7]. Furthermore, *in vitro* optimization studies demonstrated that the sequence *5*^′^*-GTAAACA-3*^′^ is preferred for FOXP3 binding to DNA [8].

The human FOXP3 gene consists of 11 exons encoding for various splice variants. Besides the full-length 431 aa variant, three truncated transcript variants lacking exon 2 (FOXP3_Δ2_), exon 7 (FOXP3_Δ7_), or both (FOXP3_Δ2,Δ7_) have been described [9,10]. However, the full-length and the Δ2 variant are the main isoforms and are equally expressed in resting human nTreg cells [10]. Generally, all isoforms share the same protein domain entities: an N-terminal repressor domain, a C2H2 zinc-finger domain, a leucine zipper domain and a C-terminal FKH domain, which also contains a nuclear localization signal. Although all known FOXP3 isoforms share the FKH domain, it is important to note that FOXP3 can also act as a co-regulator independent of the FKH binding domain [11,12].

Currently, the only contribution of FOXP3 to a full nTreg phenotype that can be defined either by gene expression patterns or on a functional level exhibiting the whole bandwidth of nTreg suppressive mechanisms is under constant controversial discussion, especially in the human system [13-15]. Nevertheless, because of their immune modulatory capability Tregs are considered key candidates for therapeutic interventions aimed at treating a broad variety of immunological diseases. If we are to better define the role of FOXP3 within nTregs, we must first gain knowledge about its condition-dependent global binding behavior, about its functional TFBSs throughout the genome, and about its competence in regulating the expression of adjacent genes.

Here we present the results of chromatin immunoprecipitation in combination with genomic tiling microarray (ChIP-on-chip) analysis of FOXP3_Δ2_, one of the main human FOXP3 isoforms, ectopically expressed in a Jurkat T-cell line under resting and mitogen-stimulated conditions. The main focus of the study was to investigate direct FOXP3/DNA interaction mediated by means of the FKH having direct impact on adjacent gene loci. The analyses revealed global distribution characteristics of human FOXP3 TFBSs and identified the cytokine IL-22 as a previously unknown direct transcriptional target of FOXP3 in activated T cells.

## Results

### Ectopically expressed FOXP3 suppresses IL-2 production in human T cells

To study FOXP3 TFBSs by ChIP-on-chip analysis, we generated a human T-cell line stably expressing FOXP3_Δ2_. Jurkat T cells were retrovirally transfected with either a viral construct expressing FOXP3_Δ2_ and green fluorescent protein (GFP) as a reporter (hereafter termed J-FOXP3) or an empty viral construct expressing only GFP (hereafter termed J-GFP). Transfected GFP^high^ T cells were sorted and subsequently expanded *in vitro*.

**Figure 1:**
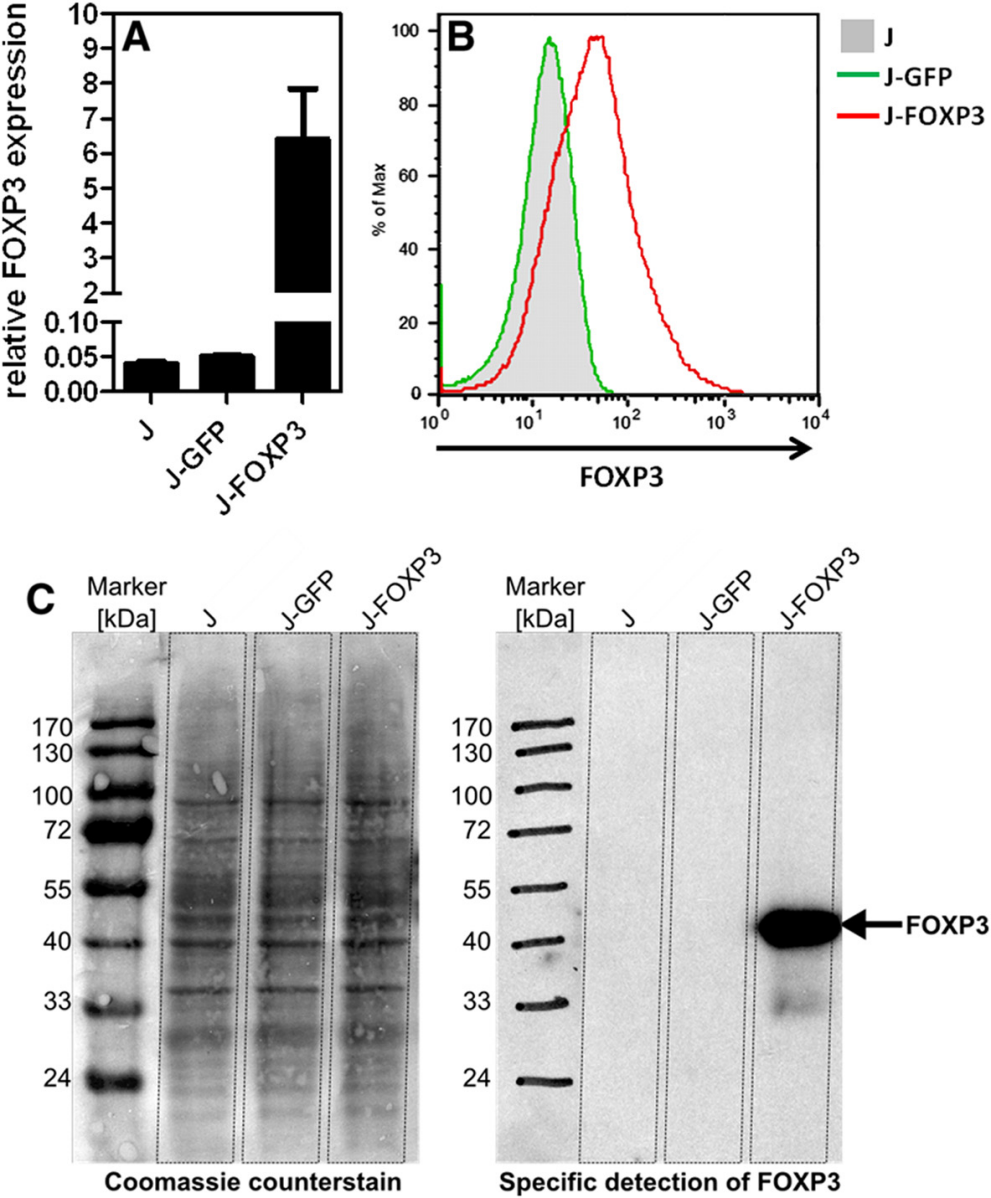
**Confirmation of FOXP3 expression in transfected Jurkat T cells.** (**A**) Quantitative real-time RT-PCR for FOXP3 expression. Bars represent mean ± SD of duplicate measurements. (**B**) FACS analysis of intracellular FOXP3 staining. (**C**) Analysis of cell lysates from equal numbers of Jurkat cells followed by FOXP3-specific Western blot analysis. All data are representative of at least two independent experiments.

FOXP3 over-expression was confirmed by quantitative real-time reverse transcriptase polymerase chain reaction (RT-PCR), flow cytometry, and Western blot analysis, as shown in Figure 1. FOXP3 mRNA levels were nearly 7 times higher in J-FOXP3 T cells than in J-GFP control cells or wild-type Jurkat T cells. Western blot analysis and intracellular FOXP3 staining also confirmed FOXP3 over-expression on the protein level exclusively in J-FOXP3 T cells.

**Figure 2:**
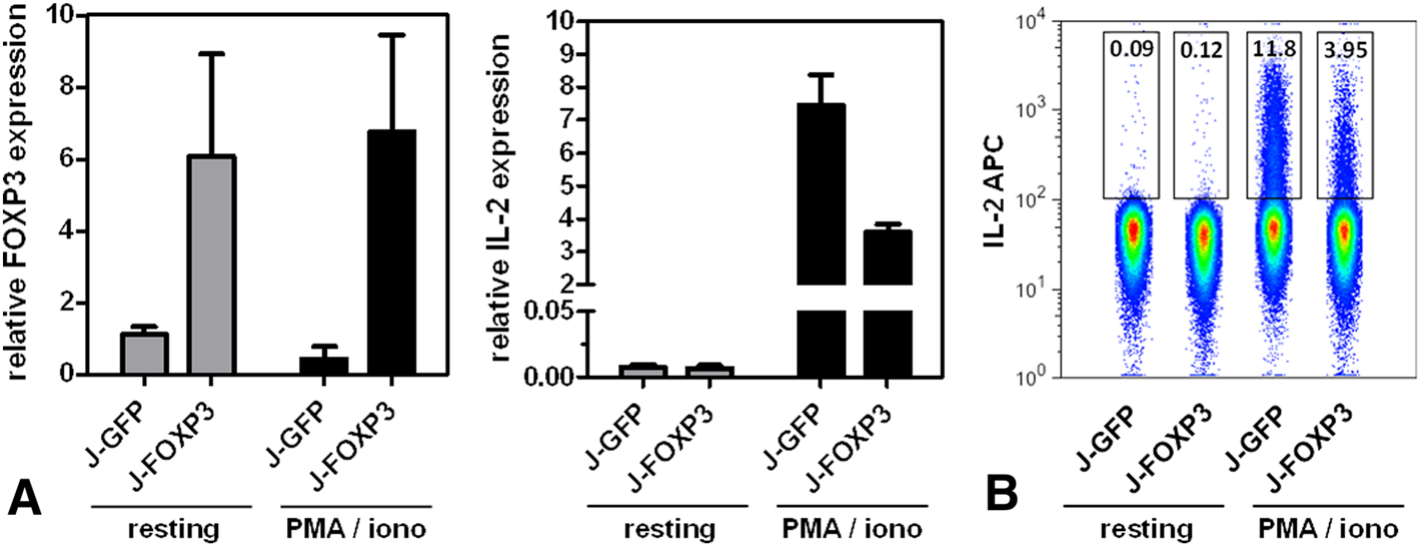
**IL-2 expression in resting and PMA/ionomycin–stimulated Jurkat cell lines.** (**A**) Quantitative real-time RT-PCR for IL-2 expression. Bars represent mean ± SD of duplicate measurements. (**B**) FACS analysis of intracellular IL-2 staining. All data are representative of at least two independent experiments.

Because interleukin-2 (IL-2) is one of the best-studied direct target genes of FOXP3, with confirmed FOXP3 binding sites in the IL-2 promoter [16], we confirmed the functionality of ectopically expressed FOXP3 protein by evaluating its influence on IL-2 production. We determined IL-2 expression by using real-time RT-PCR (Figure 2A) and intracellular cytokine staining followed by fluorescence-activated cell sorting (FACS) analysis in untreated and phorbol 12-myristate 13-acetate (PMA)/ionomycin–stimulated Jurkat T cells (Figure 2B). As expected, J-FOXP3 T cells produced less IL-2 on both the transcriptional level and the protein level than did control or wild-type cells, a finding demonstrating the ability of ectopically expressed FOXP3 to suppress IL-2 production. Taken together, the generated J-FOXP3 T cell line produced a functional FOXP3 protein and thus met an important requirement for the subsequent ChIP-on-chip studies.

### Mapping of genomic FOXP3 binding regions

It has been previously demonstrated that FOXP3 binding capability is sensitive to cellular Ca^2+^ influx and that it can be dramatically increased in T cells upon stimulation with PMA and ionomycin [16]. Therefore, we analyzed FOXP3 TFBSs in both resting and PMA/ionomycin–activated J-FOXP3 T cells in two independent replicate experiments involving a FOXP3-specific ChIP procedure, as described in Materials and Methods.

Our experiments identified 3343 ChIP regions in resting J-FOXP3 T cells and 3632 ChIP regions in stimulated J-FOXP3 T cells. More than 50% of these ChIP regions were smaller than 125 bp; this size provided a sufficient mapping resolution for the ChIP regions (data not shown). The number of ChIP regions per chromosome in general was positively correlated with the overall size of the chromosome (Pearson correlation coefficient r_resting_ = 0.91 with p = 7.07 × 10^-10^ and r_stimulated_ = 0.96 with p = 2.12 × 10^-13^; data not shown). A table of the identified FOXP3 ChIP regions and a visualized genomic map can be found in Additional File 1.

### FOXP3 preferentially binds in intronic regions located +1.6 kb from TSS

**Figure 3:**
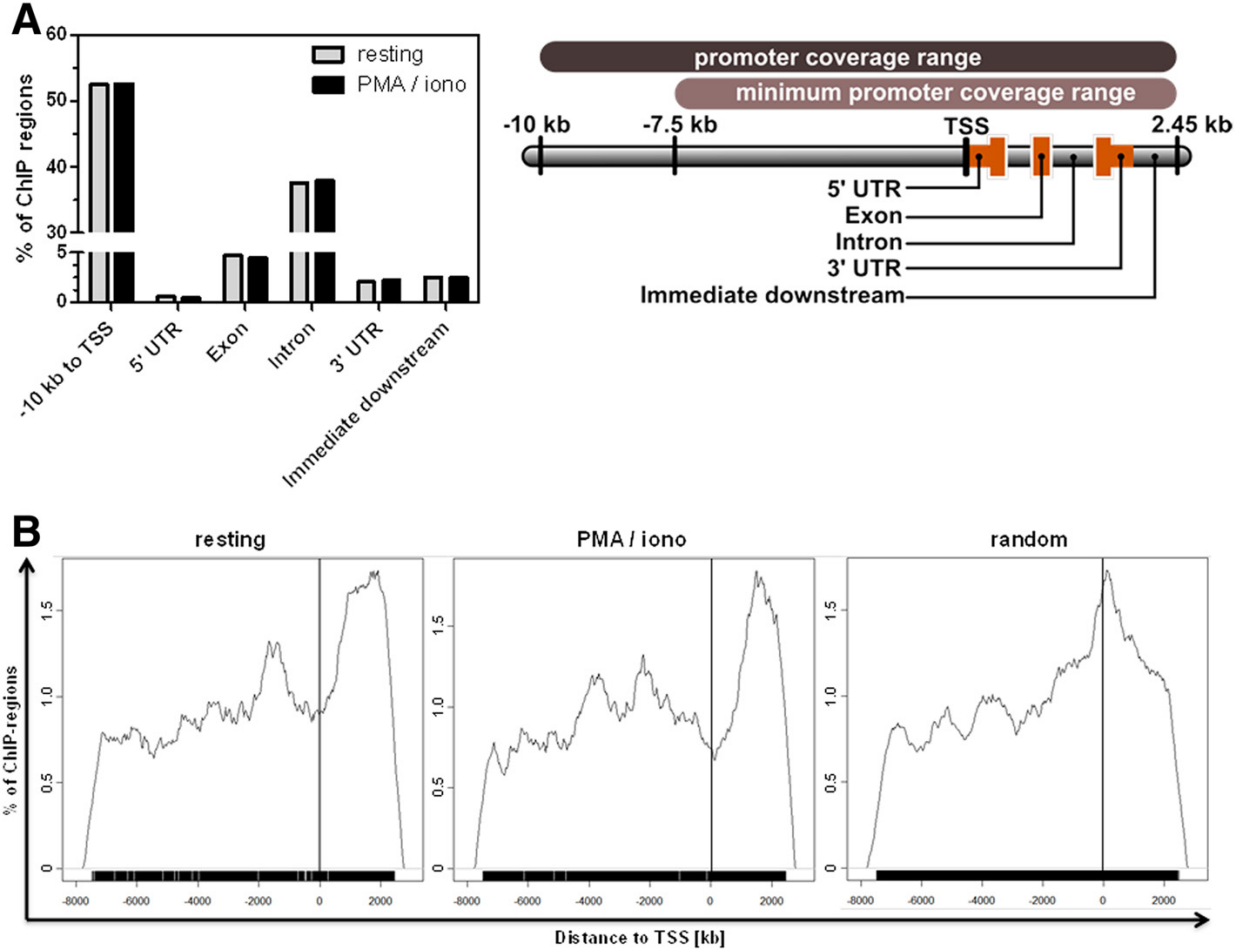
**Positional annotation of FOXP3 ChIP regions.** (**A**) CEAS annotation showing the frequency of ChIP regions in common genomic feature categories within the promoter coverage range of the tiling microarray. (**B**) Frequency of distances (in kb) of experimentally determined and randomly generated ChIP regions to their closest TSS as determined with the GPAT tool within the minimum promoter coverage range of the tiling microarray used. Bandwidth of the histograms is 172 bp.

To deduce biological meaning and more universal genome-wide characteristics of FOXP3 binding, we had to link the identified ChIP regions to known genome annotations and thus to potential FOXP3 target genes. To this end, we first used the Cis-regulatory Element Annotation System (CEAS) [17] to describe the positions of the ChIP regions in terms of known genomic feature annotation. Figure 3A gives a summary of the annotation categories provided by the CEAS database. According to the annotation, in both resting and stimulated J-FOXP3 T cells more than 50% of annotated ChIP regions are located between the transcription start site (TSS) and -10 kb. This finding was not unexpected, considering the fact that this range reflects most of the entire promoter coverage area interrogated by the tiling microarray. However, nearly 40% of annotated ChIP regions are located within an intron and thus are situated within the comparably small range from TSS to +2.45 kb. The remaining ChIP regions (~10%) fall into the positional categories: *5*^′^* untranslated region* (UTR), *Exon*, *3*^′^* UTR* and *immediate downstream*. Taken together, the findings of the CEAS annotation show that FOXP3 binds preferentially to intronic regions, independent of mitogenic stimulation.

To analyze the positional distribution of FOXP3 TFBS in reference to the TSS in more detail, we annotated the identified ChIP regions with the Genomic Position Annotation Tool (GPAT) [18]. GPAT was used in the promoter search mode, and annotation results were restricted to the tiling array’s promoter coverage range (-10 kb to +2.45 kb around the TSS). GPAT annotation resulted in 1998 annotated ChIP regions referencing 2011 gene entities in resting J-FOXP3 T cells and in 2127 ChIP regions referencing 2024 gene entities in stimulated J-FOXP3 T cells. The distances from all annotated ChIP regions to the closest TSS are presented in a histogram (Figure 3B). Interestingly, the positional distribution of ChIP regions downstream of TSS shows a marked peak at an average distance of about +1.6 kb from TSS. This main peak can be considered to result from FOXP3-bound intronic ChIP regions. However, the ChIP region distribution appears similar in both resting and stimulated conditions. A random compilation of ChIP regions matching the experimental region size and frequency distribution was further analyzed and underlined the meaning of the experiment-driven output of genomic positions for FOXP3 binding.

Pathway analysis of gene entities that were derived by GPAT annotation and that represented potential direct FOXP3 target genes was performed with GeneGO software (Thomson Reuters, New York, USA, see Additional File 2). Especially under resting conditions, the analysis identified significant (p < 0.05) enrichment of genes involved in pathways for co-stimulation of T cells, such as the CD28, CD40, ICOS, and CTLA-4 signalling pathways. Genes involved in pathways with general importance for T-cell activation, such as the NFAT, AKT, calcium and cAMP signaling pathways, were also overrepresented (p < 0.05). Of note, the IL-17 signalling pathway also appeared to be affected (p < 0.05) in the presence of FOXP3 protein.

### FOXP3 binding to certain promoter regions is associated with transcriptional changes

**Figure 4:**
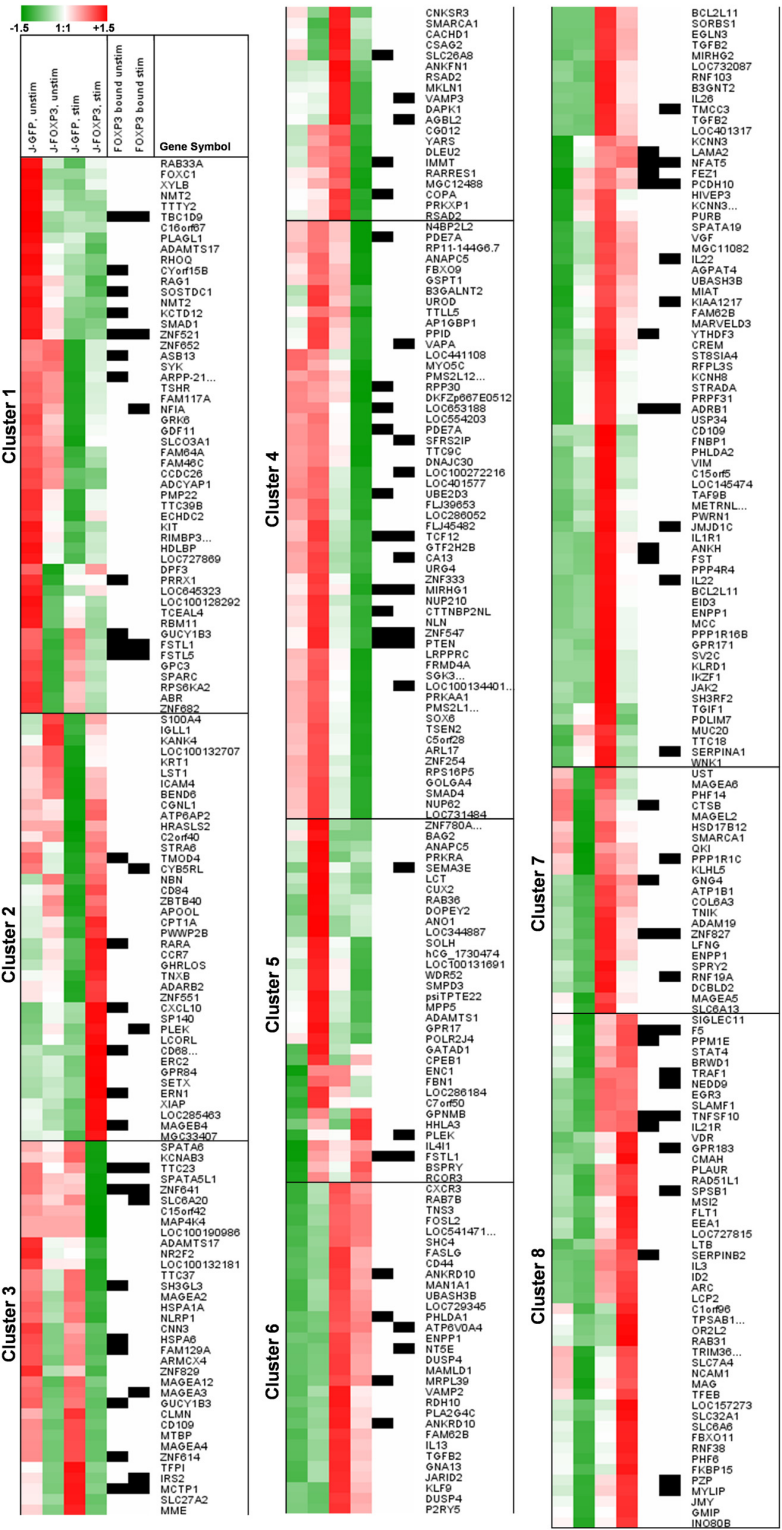
**Cluster analysis of FOXP3-dependent expressional changes in resting and PMA/ionomycin–stimulated J-FOXP3 and J-GFP cells.** Color coding represents *z*-scores of normalized expression signal intensities. Black boxes indicate the presence of at least one FOXP3 ChIP region within the transcript’s promoter region in the range of -10 kb to +2.45 kb around the TSS as determined by ChIP-on-chip analysis and GPAT annotation of resting and stimulated J-FOXP3 cells.

To link the potential competence of identified ChIP regions to FOXP3-dependent expressional changes in Jurkat T cells, we performed whole-genome expression analyses and then determined the association between expression data and promoter occupation data derived from the ChIP-on-chip studies. Expression analysis was performed on resting and PMA/ionomycin–stimulated J-GFP and J-FOXP3 T cells. Finally, we applied an analysis strategy (as described in Materials and Methods) to compile a list of 410 transcripts that exhibited FOXP3-dependent transcriptional changes. Expression data from these 410 transcripts were hierarchically clustered, resulting in 8 gene clusters. The complete set of microarray expression data is provided in Additional File 3. We combined the expression data with the GPAT ChIP region annotation list to determine the association between observed expressional changes and the binding or absence of binding of FOXP3 to the promoter region of a certain transcript. Figure 4 shows the hierarchical clustering of the 410 regulated transcripts and also indicates whether and in which experimental condition the associated gene was found in the ChIP region annotation list. Of the 410 regulated transcripts, 90 were associated with at least one ChIP hit in at least one of the two experimental conditions.

### IL-22 is a direct transcriptional target of human FOXP3 transcription factor activity

**Figure 5:**
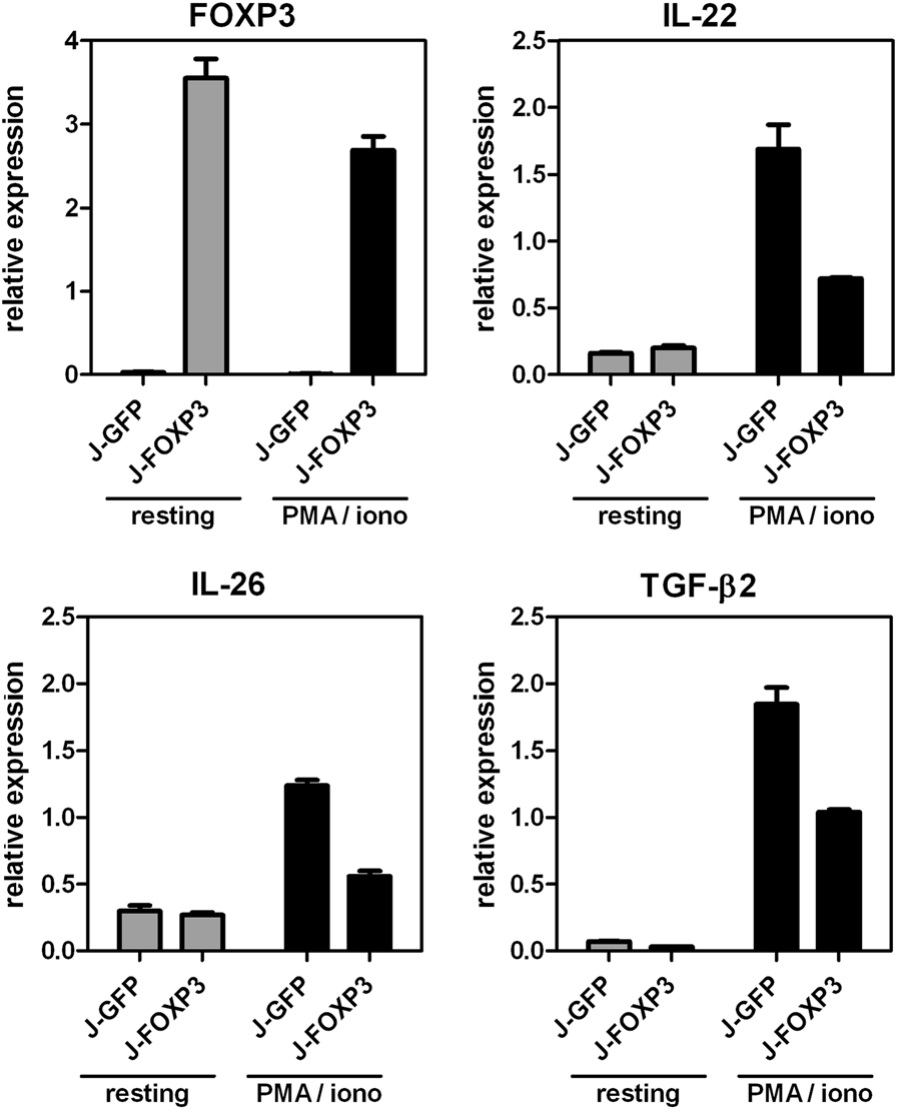
**Validation of FOXP3-dependent gene repression.** Expression of IL-22, IL-26 and TGF-β2 in resting and PMA/ionomycin-stimulated J-FOXP3 T cells by quantitative real-time RT PCR. Bars represent mean ± SD of duplicate measurements and are representative for two independent experiments.

**Figure 6:**
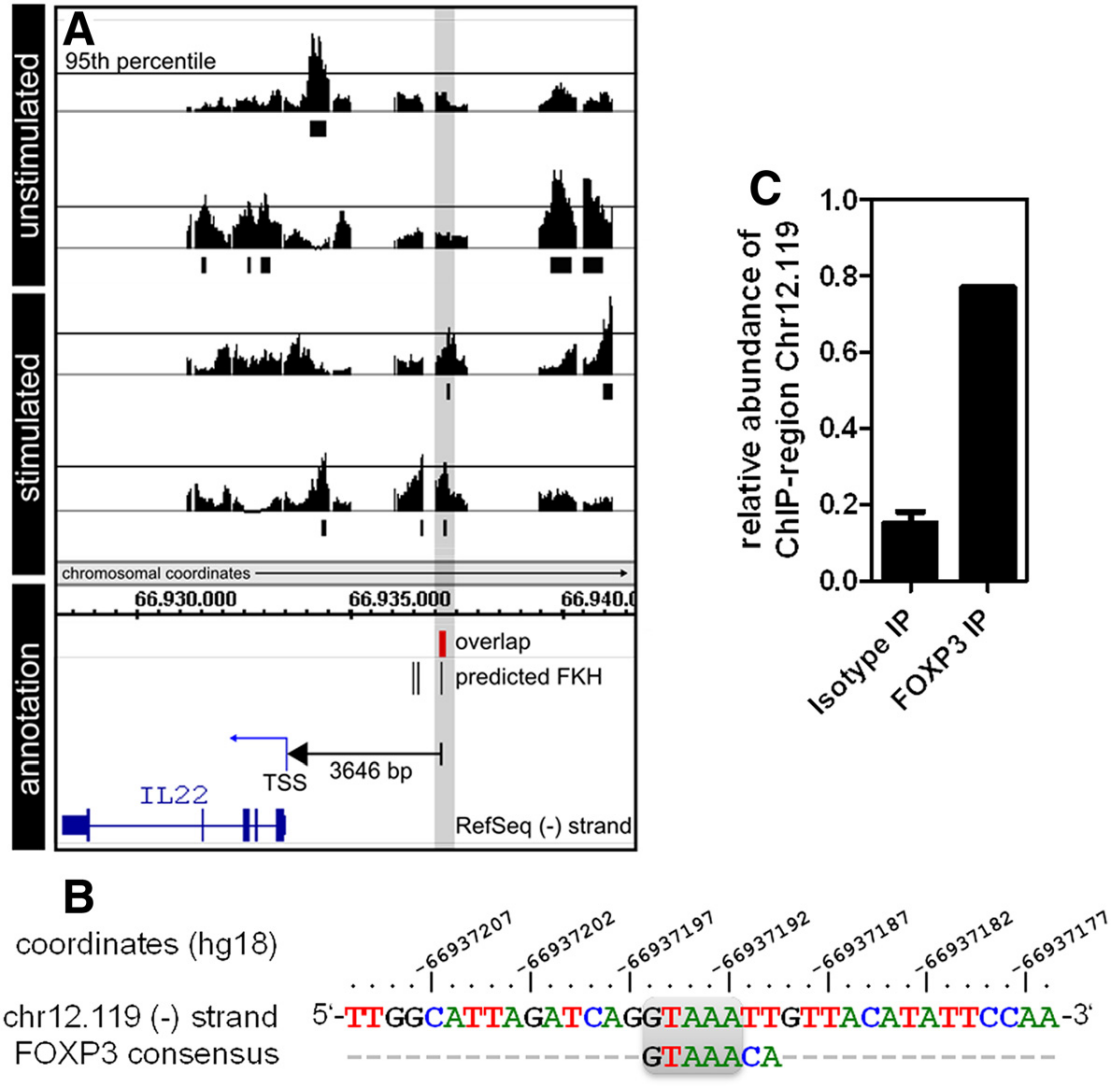
**IL-22 gene locus on chromosome 12 in more detail.** Generated ChIP-on-Chip data within the tiling array’s coverage range of the IL22 promoter alongside with RefSeq annotation. (**A**) ChIP peaks represent -10 x log_10_ of p-values derived from the local comparison of probe signal intensities from tiling microarrays of FOXP3-specific and isotype control IPs from two independent experiments using resting and PMA/ionomycin–stimulated J-FOXP3 cells. Positional overlap of ChIP regions above the p-value threshold (95th percentile) in the two replicate ChIP experiments is summarized in red below. (**B**) Sequence of summarized ChIP region chr12.119 identified in A and aligned with FOXP3 consensus sequence [8]. Chromosomal coordinates refer to hg18 genomic reference assembly. (**C**) Fold enrichment of ChIP region chr12.119 in FOXP3-precipitated J-FOXP3 material as determined by genomic quantitative real-time PCR.

In stimulated J-FOXP3 vs. J-GFP T cells, the cytokines TGF-β2 (-3.8), IL-26 (-4.0) and IL-22 (-3.8) were among the most down-regulated genes. Their FOXP3-dependent repression after treatment with PMA/ionomycin was confirmed by real-time RT-PCR (Figure 5). Interestingly, as shown in Figure 4, the observed down-regulation of IL-22 in stimulated J-FOXP3 T cells was accompanied by FOXP3 binding only under stimulated conditions. Thus, we hypothesized that IL-22 might be a stimulation-dependent direct target gene of FOXP3. Interestingly, the IL-22 genomic locus is part of a small gene cluster comprising IFN-γ, IL-26, and IL-22. The IL-22 gene lies on the minus strand of the human genome and encodes for 5 exons. Figure 6A shows the local genomic situation at the IL-22 locus in more detail.

Interestingly, a reproducible FOXP3 binding site can be found in proximity (3646 bp) to the IL-22 TSS, a finding suggesting that this site is responsible for the observed stimulation-dependent repression of IL-22 expression in PMA/ionomycin treated J-FOXP3 T cells. A prerequisite for a direct FOXP3 target gene is the occurrence of at least one FOXP3 binding site in the gene’s promoter region. Hence, under this aspect we analyzed the corresponding IL-22 promoter ChIP region (region ID: chr12.119, see Additional File 1) with a length of 36 bp. Figure 6B shows the DNA sequence of the IL-22 ChIP region on chromosome 12 and the corresponding FOXP3 FKH consensus sequence [8]. Strikingly, in the middle of the ChIP region there was a perfectly matching FOXP3 consensus site with the following genomic coordinates: chr12 66937190 – 66937197 (hg18 reference assembly). Genomic site-specific real-time PCR of this binding region confirmed about 5-fold ChIP enrichment (Figure 6C).

**Figure 7:**
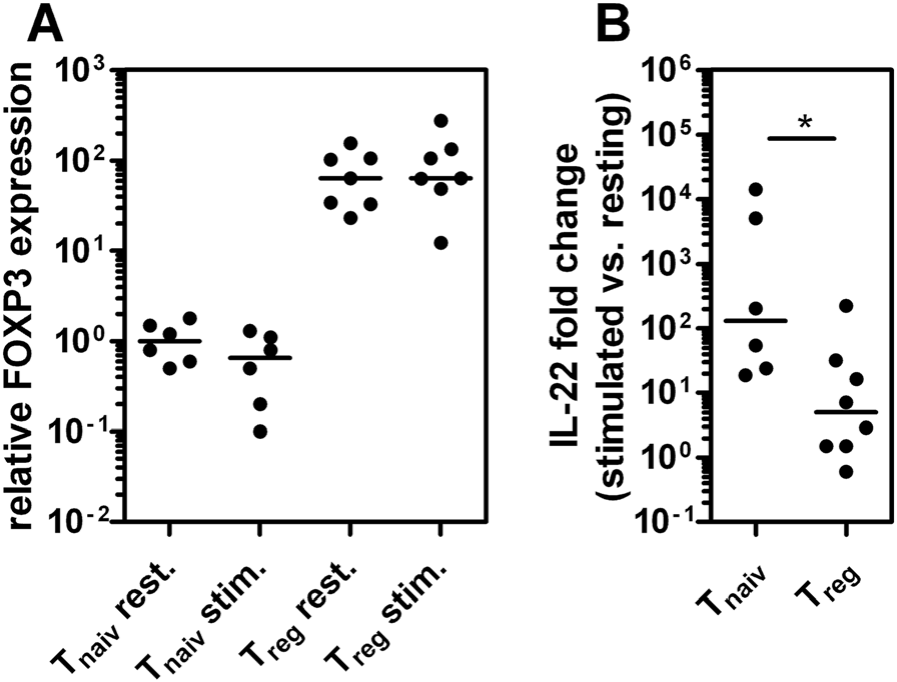
**Expression of FOXP3 and IL-22 in *ex vivo *isolated human T cells.** Human CD4^+^CD25^-^ (T_naiv_) and CD4^+^CD25^+^ (T_reg_) cells were isolated from healthy donors (n = 6-8) using AutoMACS technology and were either stimulated with PMA and ionomycin for 4 h or left untreated. Cells were subsequently analyzed by quantitative real-time RT-PCR (**A**) Expression of FOXP3 normalized to median expression of resting T_naiv_. (**B**) Fold induction of IL-22 comparing stimulated vs. resting cells. The median and individual measurements are shown. Asterisk indicates p < 0.05 in a two-sided Mann-Whitney U test.

To ultimately check whether FOXP3-dependent suppression of IL-22 following PMA/ionomycin treatment occurs in primary *ex vivo* isolated FOXP3^+^ T cells as well, CD4^+^CD25^+^FOXP3^+^ Treg cells and naïve CD4^+^CD25^-^FOXP3^-^ T cells were isolated from healthy donors using MACS technology. Following 4 h of PMA/ionomycin stimulation the cells were analyzed for FOXP3 and IL-22 expression in comparison to resting cells using real-time PCR (Figure 7). As expected, the fold induction of IL-22 expression was significantly reduced (p < 0.05) in FOXP3^+^ Treg cells compared to conventional naïve FOXP3^-^ T cells.

## Discussion

We chose to identify the functional genomic sites of direct DNA occupation by FOXP3_Δ2_ protein by using a combination of matching ChIP-on-chip and expression microarray data from human FOXP3-expressing Jurkat T cells under resting and stimulated conditions. Mitogenic stimulation with PMA and ionomycin ensures a strong simultaneous and swift T-cell activation circumventing the upstream T-cell receptor signaling, thereby allowing efficient triggering of its downstream signaling events, including activation of protein kinase C and triggering of Ca^2+^ influx, both of which are known to increase FOXP3 binding to genomic DNA [6,19]. Thus, our dataset allows us to distinguish FOXP3 binding and reversal of binding under resting conditions from alterations in FOXP3 binding behavior under stimulated conditions and to determine the direct impact on transcriptional alterations under the very same circumstances. Since in previous human FOXP3 ChIP studies by Sadlon and colleagues [7] and Bierzele and colleagues [20] only ChIP DNA from stimulated cell material has been analyzed, our dataset provides deeper insights into condition-dependent FOXP3 interaction with genomic DNA.

The tiling microarray we used interrogates proximal promoter regions of genes (basically -7.5 kb to +2.45 kb around the TSS), thereby defining comparatively narrow genomic regions predominantly with potential competence for direct transcriptional regulation of adjacent gene loci. We identified 3343 ChIP hits with proximity to 2011 GPAT-annotated gene entities under resting conditions and 3632 ChIP hits with proximity to 2024 GPAT-annotated gene entities under stimulated conditions. Of 410 markedly FOXP3-dependent transcripts, as defined by the expression microarray analysis, 90 (22%) were associated with at least one ChIP hit within the promoter coverage range of the tiling microarray. The discrepancy between the much higher number of gene entities with nearby FOXP3 ChIP hits within their promoter regions as opposed to the number of transcripts that are in fact transcriptionally altered has been observed in previous murine and human FOXP3 ChIP-on-chip studies as well [7,20-22]. This finding stresses the apparent presence of numerous silent or ineffective yet specific FOXP3 binding events in proximal promoters. However, about the biological meaning of such silent binding events can be speculated, but may for instance be explained in part by the interaction of FOXP3 with other transcription factors that may attenuate or block its functional influence on the transcription of an adjacent gene.

**Figure 8:**
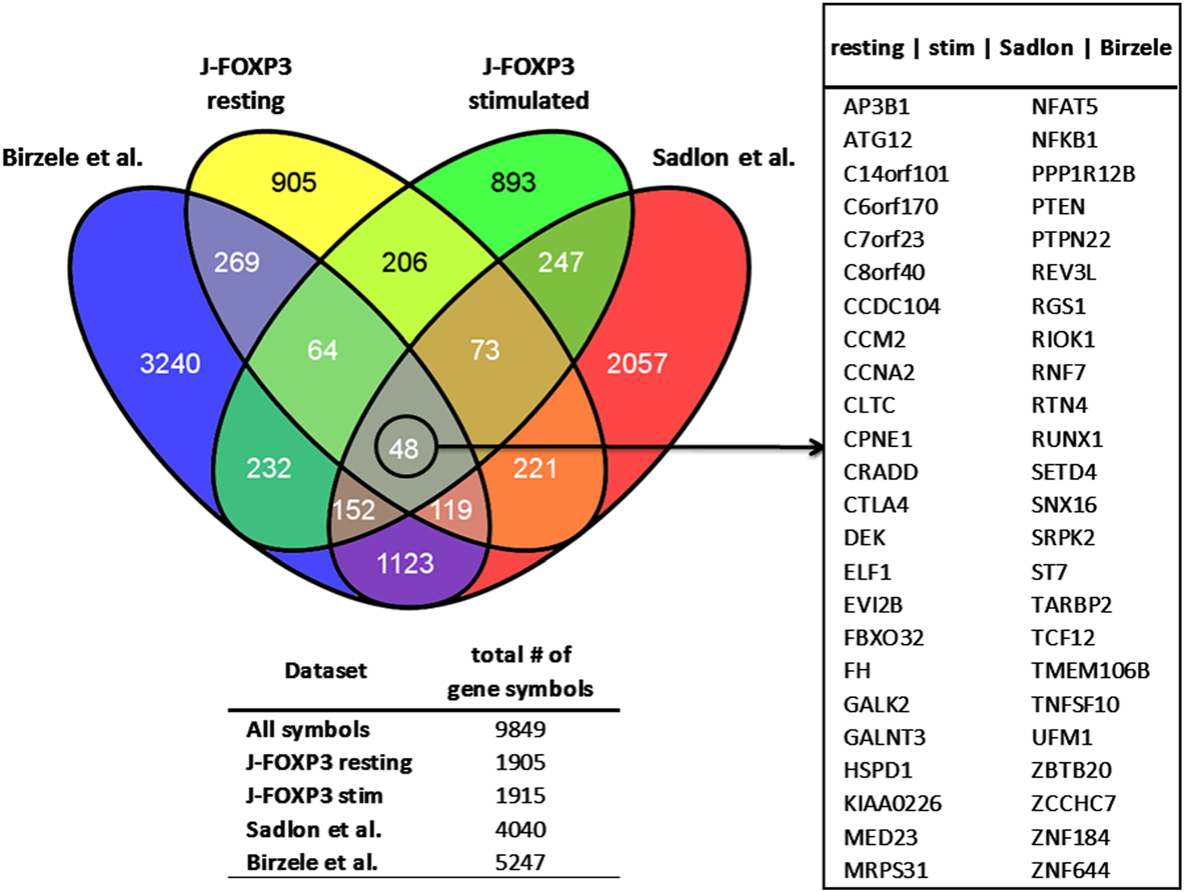
**Comparison with previously published human FOXP3 ChIP studies.** Venn diagram comparing numbers of experimentally identified FOXP3 target genes from depicted human FOXP3 ChIP studies with our findings derived from resting and stimulated J-FOXP3 cells. For comparison, all data were matched to unambiguous gene symbols from the HUGO Gene Nomenclature Committee.

To compare our annotated ChIP data to those from previous human FOXP3 ChIP studies by Sadlon [7] and Bierzele [20], which similarly provide lists of experimentally identified FOXP3 target genes, we matched all annotations to 33024 known and approved unambiguous gene symbols provided by the HUGO Gene Nomenclature Committee (HGNC) [23]. Our data represent 1905 entries in resting J-FOXP3 cells, 1915 entries in stimulated J-FOXP3 cells and 3429 combined HGNC entries (see Additional File 4). Data from Sadlon et al. resulted in a set of 4040 HGNC gene symbols, and data from Birzele et al. resulted in a set of 5247 HGNC gene symbols. Combined, both sets represent 7845 unambiguous gene symbols. Consistently, 41% (1425; p = 1.19 × 10^-132^) of the gene symbols in our dataset are also present in the combined Sadlon/Birzele gene table. Combining all four datasets separately resulted in an significant overlap of 48 loci (p = 2.25 × 10^-202^) that have been consistently identified as targets for FOXP3 binding (Figure 8). Given all technical variances that had an impact on the four datasets, e.g., the source of the cell material (*in vitro* expanded Tregs, *ex vivo* isolated Tregs, or Jurkat T cells overexpressing FOXP3) and the type of cell treatment (α-CD3/α-CD28 antibody, ionomycin, PMA/ionomycin treatment, or no treatment) and its duration (16 h, 4 h or 2 h), it is likely that these 48 genes are highly important for determining the basic Treg cell phenotype and function and are predominant targets of FOXP3 under both resting and stimulated conditions. Among these genes there are prominent ones such as *CTLA4*, *NFAT5*, *NFKB1*, *RUNX1*, and *TNFSF10*, all of which have previously been substantially associated with Treg phenotype, FOXP3 interaction, or both [24-30]. 

Both the FOXP3_Δ2_ isoform and the FOXP3_Δ7_ isoform can inhibit T-cell activation [31] and contribute to the phenotype of human nTregs [32,33]. However, FOXP3 exon 2, which encodes for a portion of the FOXP3 repressor domain, has been specifically shown to be essential for interaction with and repression of retinoic acid–related orphan receptors α and γt (RORα, RORγt) [34,35]. Both the RORα and the RORγt TFs are in turn essential for Th17 commitment of T cells [36-38]. Thus, FOXP3 expression is considered a key element preventing the establishment of a full Th17 phenotype in T cells [35].

However, in the context of T-cell plasticity some reports indicate that human RORγt^+^FOXP3^+^ Tregs exhibit the characteristics of both Tregs and Th17 cells. Consistently, it has been shown that isolated IL-17 producing FOXP3^+^ T cells that express RORγt share features of conventional RORγt^+^ Th17 cells. Interestingly, RORγt^+^FOXP3^+^ Tregs cannot sufficiently produce IL-22 after PMA/ionomycin stimulation [39]. Because we found that the presence of FOXP3 (even if exon 2 is missing) is sufficient to suppress the expression of the Th17-related cytokines IL-26 and, especially, IL-22, it is reasonable to speculate that the identified FOXP3 TFBS in the IL-22 promoter explains the inability of RORγt^+^FOXP3^+^ T cells to produce IL-22 on the transcriptional level.

## Conclusions

We successfully applied a combination of the state-of-the-art ChIP-on-chip analysis and expression microarray analysis to identify direct transcriptional targets of the human TF FOXP3_Δ2_ expressed in Jurkat T cells under resting and mitogen-stimulated conditions. Our results demonstrate that FOXP3 prefers intronic regions, independent of the cellular activation status. Notably, we found that genes important for the co-stimulation of T cells are transcriptional targets of FOXP3. In particular, we identified the Th17 lineage-related cytokine IL-22 as a previously unknown direct target gene of FOXP3. These results complete the picture of FOXP3 as a transcription factor and demonstrate its competence as a transcriptional regulator.

## Methods

### Retroviral transduction

The FOXP3_Δ2_-expressing retrovirus (pRV-FOXP3_Δ2_-IRES-GFP) and the empty control virus (pRV-IRES-GFP) were generated as previously described [40,41]. Briefly, the following primer pair was used to amplify the FOXP3_Δ2_ coding sequence from cDNA derived from human CD4^+^ T cells: 5^′^-cgggatccGGACAAGGACCCGATGCCCAACC-3^′^, 5^′^-CCCTGCCCCCACCACCTCTGC-3^′^. Retroviruses were transfected into PT67 packaging cell lines by calcium phosphate precipitation. Virus particles were produced by cultivating transfected PT67 cells for 24 h at 37°C in Iscove’s Modified Dulbecco’s Medium (IMDM) supplemented with 10% heat-inactivated fetal calf serum (FCS) and 100 U/ml penicillin/streptomycin. Remaining packaging cells were removed by filtering culture supernatants through a 0.2-μm syringe filter, after which 3 to 4 × 10^6^ Jurkat T cells were resuspended in 2.5 ml of virus supernatant in a 6-well culture plate. Next, 2.5 μl polybrene (8 mg/ml Sigma-Aldrich, Steinheim, Germany) and 50 μl HEPES (Invitrogen, Darmstadt, Germany) were added per well. Cells were centrifuged at room temperature at 500 × g for 2 h and were further incubated for 24 h at 37°C. Transduction efficiency was checked by flow cytometry, and GFP^high^ T cells were sorted with a BD FACSAria II cell sorter (Becton Dickinson [BD], Franklin Lakes, New Jersey, USA), followed by further cell expansion in IMDM.

### Isolation of primary human T cells

CD4^+^CD25^+^FOXP3^+^ and naïve CD4^+^CD25^+^FOXP3^-^ T cells were isolated from leukapheresis filters which were kindly provided by the Institute for Transfusion Medicine and Immune Hematology of the University Hospital Magdeburg. Cells were isolated using human CD4^+^CD25^+^ Regulatory T Cell Isolation Kit and AutoMACS device (both Miltenyi Biotec, Bergisch Gladbach, Germany) according to manufacturer’s instructions. Purity of isolated cells was checked by flow cytometry using CD3-FITC (clone OKT3, eBioscience, San Diego, CA, USA), CD4-PE-Cy7 (clone RPA-T4, eBioscience, San Diego, CA, USA) and CD25-PE (clone 4E3, Miltenyi Biotec, Bergisch Gladbach, Germany) antibody.

### RNA isolation

RNA was isolated by using the RNeasy Mini Kit (Qiagen, Hilden, Germany) according to the manufacturer’s recommendations. DNA was digested by using the RNase-Free DNase Set (Qiagen). RNA was eluted in 100 μl nuclease-free water and further concentrated by ethanol precipitation, beginning with the addition of 2 μl linear polyacrylamide (0.5 μg/μl,; Ambion, Darmstadt, Germany), 50 μl 7.5 M NH_4_OAc (Sigma-Aldrich), and 375 μl absolute ethanol, precooled to -20°C. RNA was precipitated for at least 2 h at -70°C and was then centrifuged at 14,000 × g at 4°C for 30 min in a table-top centrifuge. The RNA pellet was washed twice with 80% ethanol at -20°C, dried, and resuspended in nuclease-free water. RNA integrity was tested by using the Agilent 2100 Bioanalyzer (Agilent Technologies, Böblingen, Germany) with an Agilent RNA 6000 Nano/Pico Kit (Agilent Technologies).

### Real-time PCR

Equal amounts of RNA were used for cDNA synthesis in a reverse transcription reaction using a mixture of 0.5 μl oligo-dT (0.5 μg/μl) and 0.5 μl random primers (3 μg/μl) and nuclease-free water (total volume, 12 μl). Samples were incubated for 10 min at 70°C and then put on ice for 10 min. Reverse transcription (RT) reaction mix was added, containing 4 μl 5x first-strand buffer (Invitrogen), 2 μl dithiothreitol (DTT; 0.1 M, Invitrogen), 1 μl dNTP Mix (10 mM, Invitrogen), and 1 μl SuperScript II Reverse Transcriptase (Invitrogen).

Samples were incubated for 60 min at 42°C in a thermocycler. Real-time PCR was performed in duplicate with LightCycler 480 SYBR Green I Master reaction mix (Roche, Mannheim, Germany) and a LightCycler 480 system (Roche). PCR reactions were performed with 1 μl cDNA, 5 μl primer mix (containing forward and reverse primer, 500 nmol/l each), 10 μl 2x concentrated LightCycler 480 SYBR Green I Master mix (Roche), and 4 μl nuclease-free water. Relative mRNA levels were determined by using standard curves for each gene, and quantitative normalization of gene expression was performed in relation to the expression of the housekeeping gene *RPS9*. As a template for the standard curves, a mixture of all samples in 4 serial dilutions was used, corresponding to 3, 1, 0.1, and 0.01 μl template amount. The following PCR temperature cycles were used (45 cycles were performed): Step 1: 95°C, 5 min; Step 2: 95°C, 10 sec; Step 3: 55-60°C (primer dependent), 10 sec; Step 4: 72°C, 10 sec; Step 5: 4°C, hold. Real-time PCR data were analyzed with LightCycler 480 software (Roche).

Confirmation of ChIP enrichment by site-specific genomic real-time PCR was performed by using amplified ChIP DNA from FOXP3-specific immunoprecipitation and from isotype control immunoprecipitation and in reference to an unrelated genomic region within the *RPS9* gene. The following primers were used: RPS9 for: 5^′^-CGCAGGCGCAGACGGTGGAAGC-3^′^, RPS9 rev: 5^′^-CGAAGGGTCTCCGCGGGGTCACAT-3^′^, IL-2 for: 5^′^-GTCACAAACAGTGCACCTAC-3^′^, IL-2 rev: 5^′^-ATGGTTGCTGTCTCATCAGC-3^′^, FOXP3 for: 5^′^-GAACGCCATCCGCCACAACCTGA-3^′^, FOXP3 rev: 5^′^-CCCTGCCCCCACCACCTCTGC-3^′^, IL-22 for: 5^′^-CAACAGGCTAAGCACATGTCA-3^′^, IL-22 rev: 5^′^-ACTGTGTCCTTCAGCTTTTGC-3^′^, IL-26 for: 5^′^-AGCAACGATTCCAGAAGACC-3^′^, IL-26 rev: 5^′^-TGCAGTTGACCAAAAACGTC-3^′^, TGF-β2 for: 5^′^-CCAAAGGGTACAATGCCAAC-3^′^, TGF-β2 rev: 5^′^-CAGATGCTTCTGGATTTATGGTATT-3^′^, IL-22 chr12.119 for: 5^′^-AAGCCCACCTCCCAGGTCCC-3^′^, IL-22 chr12.119 rev: 5^′^-AGACAGCCAAAGCCTACTTCTGGT-3^′^

### Western blot

FOXP3 expression in transduced Jurkat T cells was detected by Western blot analysis. FOXP3 was detected by using monoclonal mouse anti-human FOXP3 antibody (clone 206D; Biolegend, San Diego, CA, USA; 1:1000 dilution in Tris buffered saline Tween 20 (TBS-T) buffer with 5% milk powder). For detection, a secondary polyclonal goat anti-mouse antibody conjugated to horseradish peroxidase (Dianova, Hamburg, Germany; 1:4000 in TBS-T buffer with 5% milk powder) was used.

### Intracellular cytokine staining

For intracellular cytokine detection, cells were cultured in IMDM medium and re-stimulated with PMA (final concentration, 10 ng/ml) and ionomycin (final concentration, 1 μg/ml) for 4 h. For the final 2 h of incubation, brefeldin A (Sigma-Aldrich) was added to block the secretion of cytokines. Subsequently, cells were fixed with 1% (v/v) paraformaldehyde solution in phosphate-buffered saline (PBS). Cells were permeabilized with 0.1% IGEPAL (Sigma-Aldrich) in PBS for 5 min. IL-2 staining was performed for 30 min (mouse anti-human IL-2 APC, clone 5344.111; BD).

### ChIP-chip procedure

Jurkat T cells were cultivated in IMDM medium supplemented with 10% FCS (PAA Laboratories) and 100 U/ml penicillin/streptomycin. If stated, cells were stimulated with PMA (10 ng/ml, Sigma-Aldrich) and ionomycin (1 μg/ml, Sigma-Aldrich) for 4 h. Protein crosslinking was ensured by the addition of formaldehyde (1% v/v) and by incubation on a shaker for 10 min at room temperature. Formaldehyde was quenched for 5 min by the addition of glycine (125 mM final concentration). After centrifugation, the culture supernatant was removed, and cells were washed once with ice-cold PBS. Cells were lysed in IP cell-lysis Buffer (10 mM Tris HCL [pH 7.5], 10 mM NaCl, 3 mM MgCl_2_, 1 mM phenylmethanesulfonyl fluoride [PMSF] 0.5% v/v IGEPAL CA-630) and incubated on ice for 10 min. Lysed cells were centrifuged at 2500 rpm for 5 min at 4°C, and the supernatant was discarded. The chromatin material was obtained by lysing the cell nuclei with IP nuclei-lysis buffer (50 mM Tris HCL [pH 8.0], 10 mM EDTA, 1% [v/v] sodium dodecyl sulfate [SDS], complete protease inhibitors) and incubating them on ice for 10 min. Chromatin was diluted in IP dilution buffer (20 mM Tris HCl [pH 8], 2 mM EDTA, 1% v/v Triton X-100, 150 mM NaCl, 1 mM PMSF) and stored at -80°C.

ChIP chromatin corresponding to 5 × 10^7^ cells was fragmented with a Branson Sonifier 250 (Branson Ultrasonics, Danbury, USA) equipped with a microtip. The following sonication conditions were applied: 60% duty cycle, maximum output power; 6 to 7 sonication cycles consisting of 30 sec on and 60 sec off. Average DNA fragment size was between 200 and 1000 bp. The IP process was performed essentially as described by the Affymetrix company: Protein G Sepharose 4B (Zymed, San Francisco, USA) and either 10 μg anti-human FOXP3 antibody (clone 206D, mouse IgG1,к; BioLegend) or 10 μg of isotype control antibody (clone MOPC-21, mouse IgG1,к; BioLegend) were used per IP, respectively.

After adapter ligation (adapter primer: 5^′^-GTTTCCCAGTCACGGTC(N)_9_-3^′^), ChIP DNA was amplified with a large-scale PCR reaction (amplification primer: 5^′^-GTTTCCCAGTCACGGTC-3^′^) involving Ampli Taq Gold DNA polymerase and partial incorporation of deoxyuridine triphosphate (dUTP). Amplified ChIP DNA was labelled and prepared for hybridization with the GeneChip WT Double-Stranded DNA Terminal Labeling Kit (Affymetrix) and was then analysed on a Human Promoter 1.0R tiling microarray (Affymetrix).

### Tiling microarray data analysis

Basic tiling array data analysis was performed with TAS software (Version 1.1.02, Affymetrix). FOXP3-bound ChIP-enriched genomic regions were identified by comparing FOXP3 IP to a matched isotype control IP from the same cell material. Only continuous genomic regions exhibiting a p-value higher than the 95th percentile of all p-values were included in subsequent analyses. Both resting and PMA/ionomycin–stimulated conditions were analyzed in independent duplicate experiments. Each replicate experiment was analyzed separately. Calculated ChIP intervals were visualized with IGB software [42]. The web-based Galaxy analysis tool [43] was used to compare repetitive ChIP-on-chip experiments. Positional intersecting ChIP regions between two replicate experiments were calculated; thus, these regions represented genomic regions that were identified in both ChIP-on-chip replicates. Moreover, the range of intersection was calculated, and only the corresponding genomic coordinates of intersecting areas were considered for further analysis. The tiling microarray data discussed in this publication have been deposited in NCBI's Gene Expression Omnibus [44,45] and are accessible through GEO Series accession number GSE37256 (http://www.ncbi.nlm.nih.gov/geo/query/acc.cgi?acc=GSE37256). Significance of the overlap between different lists of HGNC gene symbols was calculated using a hypergeometric test.

### Expression microarray analysis

Equal amounts of RNA were amplified with a double linear amplification protocol, starting with the synthesis of double-stranded cDNA by using the T7dT_23_ primer (5^′^-GGCCAGTGAATTGTAATACGACTCACTATAGGGAGGCGG(T)_23_-3^′^; Metabion, Planegg, Germany) and SuperScript II reverse transcriptase (Invitrogen, Karlsruhe, Germany). A second-strand cDNA synthesis was then performed with DNA polymerase I (Invitrogen) and *E. coli* DNA ligase (Invitrogen). After purification of double-stranded cDNA, the first amplification round was performed in an *in vitro* transcription reaction by using the Promega P1300 RiboMax Kit for T7 amplification (Promega, Mannheim, Germany); this procedure produced unlabeled cRNA. The amplified cRNA was subjected to a second amplification round, starting again with reverse transcription by using random hexamer primers (Pharmacia, Freiburg, Germany) for first-strand synthesis of cDNA, followed by cRNA degradation with RNase H treatment. T7dT_23_ primers were again used for the second-strand synthesis, as described above. In the second *in vitro* transcription, which used the GeneChip expression 3^′^-Amplification Reagent Kit for labeling (Affymetrix, San Francisco, CA, USA), biotinylated UTP was partially incorporated into the final cRNA. The quantity and quality of biotinylated cRNA were checked photometrically. Biotinylated cRNA was fragmented, hybridized to a Human Genome U133 Plus 2.0 Array (Affymetrix), washed, and stained as recommended by the manufacturer. Microarray data were analyzed with GeneSpring GX 10.0 software (Agilent Technologies) and the Robust Multi-array Analysis (RMA) normalization algorithm. Cluster analysis was performed with Genesis Software 1.7.3 [46] by using a *z*-score transformation [47]. A list of 410 FOXP3-dependent genes was compiled by combining two analysis strategies. First, J-FOXP3 cells were compared with J-GFP cells. Genes were considered to be influenced by the over-expressed FOXP3 protein only if their expression showed at least a two-fold change (up or down) under either stimulated or unstimulated conditions. In a second strategy, stimulated and resting J-FOXP3 and stimulated and resting J-GFP cells were compared by calculating the differences in gene expression. Only genes that showed at least a two-fold change in stimulation-dependent induction or repression were considered to be influenced by the over-expressed protein. Subsequently this list of stimulation-dependent transcripts was filtered for differences between FOXP3-expressing cells and GFP control cells. Only transcripts that exhibited at least a two-fold difference in stimulation dependency between J-FOXP3 and J-GFP cells were retained. Both analysis strategies resulted in a combined list of 410 FOXP3-dependent genes. The expression microarray data discussed in this publication have been deposited in NCBI's Gene Expression Omnibus [44,45] and are accessible through GEO Series accession number GSE37256 (http://www.ncbi.nlm.nih.gov/geo/query/acc.cgi?acc=GSE37256).

## Abbreviations

ChIP: Chromatin immunoprecipitation; FKH: Forkhead; FOXP3: Forkhead box P3; GPAT: Genomic Position Annotation Tool; Iono: Ionomycin; IP: Immune precipitation; MACS: Magnetic activated cell sorting; PMA: Phorbol 12-myristate 13-acetate; TF: Transcription factor; TFBS: Transcription factor binding site; Treg: Regulatory T cell; TSS: Transcription start site.

## Competing interests

The authors declare that they have no competing interests

## Authors’ contributions

AJ performed the experiments and data analyses. WH designed, cloned, and provided the retroviruses. FE performed cell isolation and quantitative real-time PCR. JB initiated the study and provided conceptual ideas. RG was principal investigator and was involved in all microarray experiments and data analysis. DB was principal investigator and wrote the manuscript together with AJ. All authors read and approved the final manuscript.

## Supplementary Material

Additional File 1 **Identified FOXP3 ChIP-regions.** List of all identified FOXP3 ChIP regions in resting and PMA/ionomycin-stimulated FOXP3-over-expressing Jurkat T cells including chromosomal coordinates, a visual representation map of their chromosomal distribution and their genomic annotation according to the Genomic Position Annotation Tool. Click here for file

Additional File 2 **GeneGO pathway analysis of FOXP3-bound genes.** Analysis of annotated FOXP3 ChIP hits for statistically over-represented pathways. Click here for file

Additional File 3 **Expression microarray data.** List of 410 transcripts responding to FOXP3-over-expression in Jurkat T cells under resting and PMA/ionomycin-stimulated conditions. Click for file

Additional File 4 **Comparison of human FOXP3-ChIP studies.** Table containing comparison of identified FOXP3 ChIP hits with previously published human FOXP3-ChIP data. Click here for file
