# Supplementary material for: ChIP-on-chip analysis identifies IL-22 as direct target gene of ectopically expressed FOXP3 transcription factor in human T cells
Source: BMC Genomics. 2012 Dec 17;13:705. doi: 10.1186/1471-2164-13-705 (PMC3547697; doi:10.1186/1471-2164-13-705)
Supplement: Additional File 2 — GeneGO pathway analysis of FOXP3-bound genes. Analysis of annotated FOXP3 ChIP hits for statistically over-represented pathways. [file 1471-2164-13-705-S2.pdf]

Category	Pathway	resting cells (FDR adjusted p-value*)	stimulated cells (FDR adjusted p-value*)	ChIP hits	Molecules in complete pathway	Ratio
**Apoptosis and survival**	APRIL and BAFF signaling	0.331900	**0.000526**	8	38	21.1%
	BAD phosphorylation	**0.000302**	0.059930	12	42	28.6%
	Caspase cascade	0.097170	**0.001119**	10	33	30.3%
	Ceramides signaling pathway	**0.003542**	0.303600	9	38	23.7%
	FAS signaling cascades	0.195900	**0.000248**	11	43	25.6%
	HTR1A signaling	**0.004630**	0.003386	12	50	24.0%
	NO synthesis and signaling	**0.002322**	0.058840	12	55	21.8%
**Cancer**	Hypoxia-induced EMT in cancer and fibrosis	0.390200	**0.000673**	5	9	55.6%
	PGE2 pathways in cancer	**0.002322**	0.145500	12	55	21.8%
	Role of alpha-6/beta-4 integrins in carcinoma progression	**0.002347**	0.193300	11	45	24.4%
	Some pathways of EMT in cancer cells	0.289700	**0.000933**	12	51	23.5%
**Cardiac Hypertrophy**	NF-AT signaling in Cardiac Hypertrophy	**0.000585**	0.005242	17	65	26.2%
**Cell adhesion**	Chemokines and adhesion	**0.000815**	0.004555	19	100	19.0%
	Ephrin signaling	**0.000520**	**0.000356**	15	45	33.3%
	PLAU signaling	**0.004123**	0.317700	9	39	23.1%
**Cell cycle**	Regulation of G1/S transition (part 1)	**0.003542**	0.011460	9	38	23.7%
**Cytoskeleton remodeling**	Role of PKA in cytoskeleton reorganisation	**0.001058**	0.143300	10	40	25.0%
**Development**	Angiopoietin - Tie2 signaling	**0.002167**	0.099500	9	35	25.7%
	Angiotensin activation of Akt	**0.002712**	0.415900	9	46	19.6%
	Delta- and kappa-type opioid receptors signaling via beta-arrestin	0.031770	**0.000797**	7	23	30.4%
	Delta-type opioid receptor mediated cardioprotection	**0.003026**	0.289500	8	37	21.6%
	EGFR signaling via PIP3	**0.001044**	0.005184	8	23	34.8%
	Endothelin-1/EDNRA signaling	**0.001779**	0.004897	13	53	24.5%
	FGF2-dependent induction of EMT	**0.003388**	0.647700	6	20	30.0%
	Gastrin in differentiation of the gastric mucosa	**0.003542**	0.303600	9	38	23.7%
	Leptin signaling via PI3K-dependent pathway	**0.000149**	0.030460	12	47	25.5%
	Ligand-independent activation of ESR1 and ESR2	**0.002022**	0.070550	10	44	22.7%
	MAG-dependent inhibition of neurite outgrowth	0.133300	**0.000434**	10	37	27.0%
	Melanocyte development and pigmentation	**0.000996**	0.101300	11	49	22.4%
	NOTCH1-mediated pathway for NF-KB activity modulation	0.105700	**0.001348**	7	34	20.6%
	PEDF signaling	**0.000996**	0.002973	13	49	26.5%
	PIP3 signaling in cardiac myocytes	**0.000027**	0.030460	14	47	29.8%
	Regulation of epithelial-to-mesenchymal transition (EMT)	**0.001934**	**0.000329**	18	64	28.1%
	Role of CDK5 in neuronal development	**0.001816**	0.521100	9	34	26.5%
	Role of HDAC and calcium/calmodulin-dependent kinase (CaMK) in control of skeletal myogenesis	**0.000106**	0.005500	17	54	31.5%
	S1P1 signaling pathway	**0.002022**	0.070550	11	44	25.0%
	S1P3 receptor signaling pathway	**0.000364**	**0.001247**	14	43	32.6%
	Thrombopoietin-regulated cell processes	0.031330	**0.001697**	11	45	24.4%
	VEGF signaling and activation	**0.001734**	0.065120	10	43	23.3%
**DNA damage**	Inhibition of telomerase activity and cellular senescence	**0.000460**	0.270000	6	20	30.0%
**G-protein signaling**	Proinsulin C-peptide signaling	**0.001548**	0.004345	14	52	26.9%
	RhoA regulation pathway	0.105700	**0.000035**	12	34	35.3%
	S1P2 receptor signaling	**0.002167**	0.007654	10	35	28.6%

Category	Pathway	resting cells (FDR adjusted p-value*)	stimulated cells (FDR adjusted p-value*)	ChIP hits	Molecules in complete pathway	Ratio
**Immune response**	CD28 signaling	**0.000493**	0.005500	14	54	25.9%
	CD40 signaling	**0.001934**	0.015080	14	64	21.9%
	Fc epsilon RI pathway	**0.002322**	0.020420	12	55	21.8%
	Fc gamma R-mediated phagocytosis in macrophages	**0.002347**	0.674200	9	45	20.0%
	Function of MEF2 in T lymphocytes	**0.004630**	0.012470	14	50	28.0%
	ICOS pathway in T-helper cell	**0.002712**	0.027710	12	46	26.1%
	IL-17 signaling pathways	**0.000063**	0.010390	15	60	25.0%
	NFAT in immune response	**0.001342**	0.013850	12	51	23.5%
	PGE2 common pathways	**0.001548**	0.270300	11	52	21.2%
	PIP3 signaling in B lymphocytes	**0.001479**	0.059930	11	42	26.2%
	Regulation of T cell function by CTLA-4	**0.000085**	0.033920	11	36	30.6%
**Metabolism**	GTP metabolism	**0.002036**	**0.000012**	18	54	33.3%
**Muscle contraction**	Oxytocin signaling in uterus and mammary gland	**0.004265**	0.187600	13	60	21.7%
	Regulation of eNOS activity in endothelial cells	**0.001934**	0.015080	16	64	25.0%
	Relaxin signaling pathway	0.111600	**0.000589**	12	48	25.0%
	Role of kappa-type opioid receptor in heart	**0.001510**	0.005688	10	33	30.3%
**Neurophysiological process**	ACM regulation of nerve impulse	0.010410	**0.000423**	13	46	28.3%
	Dopamine D2 receptor transactivation of PDGFR in CNS	**0.000322**	0.008943	9	26	34.6%
	Receptor-mediated axon growth repulsion	0.090410	**0.001697**	11	45	24.4%
**Protein folding**	Membrane trafficking and signal transduction of G-alpha (i) heterotrimeric G-protein	**0.000337**	**0.000002**	11	19	57.9%
**Proteolysis**	Role of Parkin in the Ubiquitin-Proteasomal Pathway	**0.001330**	**0.001018**	10	24	41.7%
**Signal transduction**	Activation of PKC via G-Protein coupled receptor	**0.001548**	**0.000237**	17	52	32.7%
	AKT signaling	**0.000364**	0.005451	12	43	27.9%
	Calcium signaling	**0.002347**	0.076230	13	45	28.9%
	cAMP signaling	**0.000739**	0.002655	12	38	31.6%
**Transcription**	ChREBP regulation pathway	**0.000615**	0.289200	6	21	28.6%
	CREB pathway	**0.000014**	0.070550	12	44	27.3%
	P53 signaling pathway	0.153200	**0.000632**	9	39	23.1%
	Sin3 and NuRD in transcription regulation	**0.003542**	0.303600	9	38	23.7%
**Translation**	Non-genomic (rapid) action of Androgen Receptor	**0.001058**	0.014630	10	40	25.0%
**Transport**	Macropinocytosis regulation by growth factors	**0.000443**	0.039530	16	63	25.4%

*** p-values in bold: FDR < 0.05 %**
